# *In Silico* Frontiers Shaping the Next Generation of Transformation Product Prediction and Toxicological Assessment

**DOI:** 10.1021/acs.est.5c06790

**Published:** 2025-09-02

**Authors:** Paul Löffler, Emma L. Schymanski, Henning Henschel, Foon Yin Lai

**Affiliations:** † Department of Aquatic Sciences and Assessment, 8095 Swedish University of Agricultural Sciences (SLU), P.P. Box 7050, SE-750 07 Uppsala, Sweden; ‡ Luxembourg Centre for Systems Biomedicine (LCSB), University of Luxembourg, 4367 Belvaux, Luxembourg; § Department of Medicinal Chemistry, Uppsala University (UU), Uppsala SE-751 21, Sweden

**Keywords:** environmental fate, computational (eco)toxicology, chemical prioritization, risk assessment, QSAR modeling, rule-based models, machine learning, organic micropollutants

## Abstract

The characterization of transformation products (TPs) is crucial for understanding chemical fate and potential environmental hazards. TPs form through (a)­biotic processes and can be detected in environmental concentrations comparable to or even exceeding their parent compounds, indicating toxicological relevance. However, identifying them is challenging due to the complexity of transformation processes and insufficient data. *In silico* methods for predicting TP formation and toxicity are efficient and support prioritization for chemical risk assessment, yet require sufficient data for improved results. This perspective article explores the role of computational approaches in assessing TPs and their potential effects, including rule-based models, machine learning-based methods, and QSAR-based toxicity predictions, focusing on openly available tools. While integrating these approaches into computational workflows can support regulatory decision-making and prioritization strategies, predictive models can face limitations related to applicability domains, data biases, and mechanistic uncertainties. To better communicate the results of *in silico* predictions, a framework of four distinct levels of confidence is proposed to support the integration of TP prediction and toxicity assessment into computational pipelines. This article highlights current advances, challenges, and future directions in applying *in silico* methodologies for TP evaluation, emphasizing the need for more data and expert interpretation to enhance model reliability and regulatory applicability.

## Graphical abstract

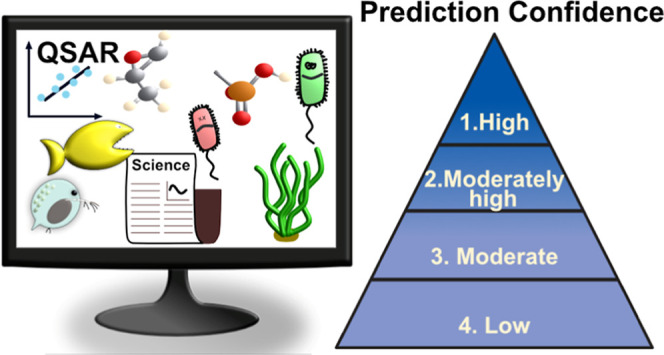

## 1 Introduction

The importance of characterizing transformation products (TPs) potentially affecting the receiving aquatic environments has been increasingly emphasized,^1–4^ with many TPs found in similar or even higher environmental concentrations than their respective parent compound.^5–7^ For example, Kołecka et al. quantified two diclofenac TPs in effluent wastewater with concentration levels almost double than diclofenac itself.^8^ However, discovering all possible TPs is challenging. Several Organization for Economic Co-operation and Development (OECD) guidelines exist to investigate environmental (e.g., photo, microbial) transformation of chemicals in aquatic ecosystems.^9–12^ This perspective considers TPs from multiple transformation pathways, including abiotic processes such as photolysis or water treatment, and biotic processes such as environmental biotransformation and human metabolism. TPs formed within living organisms (i.e., metabolites or biotransformation products) can be identified via *in vitro* or *in vivo* methods. The former involves exposure of a chemical to specific enzymes in laboratory-scale experiments, while the later refers to the analysis of biological matrices, such as blood, tissue, or excreta, following exposure to a chemical. Complicating factors in these methods include ethical considerations and the variability across different organisms and environmental contexts.^13–19^ TPs formed through abiotic reactions such as photolysis or treatment processes can be determined through laboratory experiments or pilot plants, with sophisticated setups.^20–26^ The analytical method of choice for identifying and discovering new TPs is high-resolution mass spectrometry (HRMS), generating extensive datasets that require careful investigation to accurately identify each TP, with many features remaining unidentified or only tentatively identified.^3,27,28^ Several TPs have been shown to contribute to the overall hazard and risk profile in the environment.^29–36^ For example, fluoxetine,^37^ propranolol,^38^ and acyclovir^3^ TPs have been suggested to exhibit (eco-)­toxicological effects. Recently, 6PPD-quinone, the TP of the tire additive 6PPD (4-*N*-(4-methylpentan-2-yl)-1-*N*-phenylbenzene-1,4-diamine) that can enter environmental waters through for example urban runoff, was shown to exhibit toxic effects to multiple fish species, with toxicity levels several orders of magnitude higher than 6PPD itself.^30,39–43^ Given the established contribution of several TPs to the overall hazard and risk profile of environmental samples, a holistic risk assessment aims at covering as much of the chemical space as possible. However, it is neither practical nor realistic to assess risks of all potential chemicals and their TPs individually through HRMS and ecotoxicological studies.

Combining chemical with effect-based methods and *in silico* approaches has been suggested to investigate combined effects and mechanisms of toxicity.^44–46^ *In silico* methodologies can help to fill knowledge gaps and support screening or prioritization. Computational approaches can predict how chemicals would behave in the environment and their potential toxic effects, including quantitative structure activity relationships (QSARs) and read-across methods.^47,48^ Additionally, molecular docking and molecular dynamics simulations, widely used in medicinal chemistry, are increasingly considered for chemical safety assessments, offering potential insights into toxic action mechanisms.^46^ Comprehensive workflows can now predict TPs and key toxicological endpoints from just the initial chemical structure. Such approaches could serve as essential safety measures, for example, in early assessment stages for regulatory and drug design purposes, enabling more informed decision-making in chemical production. Additionally, these methodologies allow for the integration of TP assessments, aiding environmental scientists and other stakeholders in managing chemical impacts effectively.

This perspective article explores how *in silico* methodologies can enhance the risk assessment process for TPs in order to facilitate the development of computational workflows that integrate TP formation and toxicity assessments. This could be beneficial to various fields, including pharmaceutical development and environmental sciences, by enabling proactive evaluations of chemical safety and environmental impacts. The motivation stems from recent recommendations within the scientific community for early integration of persistence and toxicity measures into management frameworks to implement a more proactive approach.^49–51^ This article focuses on broadly applicable open access *in silico* approaches for predicting TPs and toxicological impacts. Tools are compared based on their functionality, input requirements, applicability domain, interpretability, and validation strategies. This work also highlights emerging computational approaches, current challenges, and research needs in TP prediction and toxicological assessment.

## 2 Foundations of Predictive Approaches

There are two primary computational approaches: rule-based models and machine learning-based models, each with strengths and limitations, offering complementary insights into chemical behavior and risks.

### 2.1 Rule-Based Models

Rule-based models are grounded in mechanistic evidence derived from experimental studies. They rely on predefined rules or structural alerts, molecular substructures or patterns associated with specific biological activities, transformations, or toxicological endpoints. In TP prediction, rule-based models apply expert-curated reaction rules to forecast transformations such as hydroxylation or oxidation. In toxicology, the presence of a structural alert, such as a nitro group linked to mutagenicity,^52^ can serve as indicator for hazard identification. The interpretability of rule-based models is one of their key strengths, as they are built on well-defined reaction pathways or mechanistic insights. However, they are inherently constrained by the width and depth of their underlying libraries. This means they can only predict behaviors and transformations/mode of actions that have already been characterized, limiting their utility for novel chemicals or uncharted mechanisms.

### 2.2 Machine Learning Models

Machine learning (ML) models are data-driven and particularly effective in capturing complex, nonlinear relationships. By analyzing large datasets of chemical properties, structures, and biological activities, these models can uncover patterns and make predictions that extend beyond existing mechanistic knowledge.^53^ In TP prediction, ML algorithms can predict potential transformation pathways based on chemical descriptors and environmental factors. In toxicological assessment, ML models can estimate effects like bioaccumulation or endocrine activity by learning from extensive experimental datasets. While ML models are powerful and flexible, their reliability depends on the quality, diversity, and size of the training datasets. They also face challenges like overfitting, where the model performs well on training data but poorly on unseen data. Additionally, the black-box nature of many ML methods can hinder interpretability, making it difficult to trace predictions back to mechanistic insights.

### 2.3 Integration and Complementarity

Rule-based and ML models are not mutually exclusive but complementary. Workflows and approaches that integrate both these approaches combine the reliability of expert knowledge with the adaptability of data-driven insights. QSAR models serve as a bridge between rule-based and ML approaches, as they can be developed using expert-defined descriptors rooted in mechanistic knowledge or trained on large datasets using statistical learning methods. Similarly, read-across approaches, which involve predicting properties of a target chemical using data from structurally similar, well-studied analogues, are increasingly enhanced by ML to improve predictive accuracy.^54,55^ This combined approach forms the foundation of predictive methodologies discussed in the following sections, illustrating how these techniques are applied.

## 3 Finding Data on Known Transformation Products

Datasets of known TPs are the starting point for most investigations and form the basis for developing rule-based and ML approaches discussed above. Systematic literature searching (e.g., predefining specific search strings and using multiple scientific databases) usually results in a large number of articles that need to be screened. Multiple text-mining tools^56–59^ assist and facilitate this work, including chemical data extraction pipelines.^60–62^ ShinyTPs was specifically designed to curate TP information derived from text-mining of hand-selected text snippets integrated within PubChem.^62^ With increased contribution to and awareness of open access TP resources, such as enviPath^63,64^ and suspect lists on the NORMAN Suspect List Exchange (NORMAN-SLE),^65^ screening existing databases^66^ or shared suspect lists for TPs^67–82^ has become more common. Several lists with parent-TP mappings on the NORMAN-SLE^65^ have been mapped up into transformations templates,^83^ added into PubChem in the “Transformations” section and archived as an (updatable) data set on Zenodo.^66^ This enables both public display (in PubChem) to raise awareness of the data, and integration into TP identification workflows, such as those integrated within patRoon.^21,84,85^ This collaborative community effort currently includes 9152 unique reactions involving 9267 unique compounds. Of the chemicals included, 3724 are classified as parents and 7331 as TPs (some are both parent and TPs in different reactions). Although these numbers have grown considerably in the last years and are now triple what was used to train BioTransformer^86,87^ (detailed further below), this is still a tiny fraction (<0.1%) of the currently >131 000 compounds in the NORMAN-SLE,^65^ and an even smaller fraction (<0.0001%) of the chemicals in PubChem. The lack of sufficiently documented open data on TPs is a huge challenge for establishing reliable computational methods, as the current knowledge focuses on only certain chemical classes in great detail, yet does not cover many other classes that are known to be present in these databases.

While it is feasible that large language models (LLMs), such as ChatGPT, can be prompted to propose lists of possible TPs, they should be treated with caution, as their outputs are not based on curated chemical reaction rules or mechanistic understanding, and assessing their applicability domain is currently not feasible. To date, systematic exploration or scientific validation of LLMs for TP prediction is lacking. In-depth analysis and prediction using LLMs is therefore not recommended, as they can often generate plausible-sounding but false or unverifiable information.^88,89^ In contrast, databases documenting known TP reactions offer a higher level of reliability and transparency, as they provide carefully curated data by experts following strict criteria for data inclusion and referencing protocols for verification, ensuring a more trustworthy source of information.

## 4 Prediction of Transformation Products

*In silico* strategies that predict TPs using expert knowledge or pattern recognition for the creation of suspect lists for improved screening in HRMS experiments have gained attention.^28^ These computational tools are valued for their ability to generate novel chemical structures, whether plausible or not. The *in silico* TP prediction tools discussed in this work incorporate a comprehensive array of underlying transformation rules and models, tailored for diverse processes such as phase I or phase II metabolism, and environmental microbial degradation. With increasing attention to advanced treatment technologies, it is feasible that these approaches could be expanded to cover such transformation reactions as more data on TPs from advanced treatment processes becomes available. To support these advancements, it is crucial that researchers share experimental data on transformation reactions, to enhance model development and validation. The ACS author guidelines for several environmental journals have recently been updated to provide some instructions and suggestions to authors how to share this information.^90^ Unless otherwise specified, the tools discussed below are limited to organic compounds under ∼1000–1500 Da, and do not support polymers, nanomaterials, or highly fluorinated substances due to a lack of representative training data or rules.

BioTransformer, an open source tool, includes eight models of metabolic transformation prediction, including phase I (cytochrome P450), promiscuous enzymatic, phase II, human gut microbial, environmental microbial transformations and different combinations of the above known as *AllHuman*, *SuperBio* and *MultiBio*.^86,87^ Users can submit molecular structures as Simplified Molecular-Input Line-Entry System (SMILES), a line notation describing chemical structures, or as a Structured Data File (SDF), a standard format for storing molecule structure information and associated data. BioTransformer is available as command-line tool and through a web server at www.biotransformer.ca. While it supports batch processing of chemicals, it does not allow for batch mode across multiple models. However, this limitation can be overcome using the command line version and a bash script (example file and explanation can be found here: https://github.com/paloeffler/biotrans_multiprompt) that loops over all the models of interest. The web tool outputs an interactive table of the predicted TPs. An example of antimicrobial TPs generated via BioTransformer and the mentioned script is published online in NORMAN suspect list S114.^82^ BioTransformer integrates rule-based and ML approaches, and its underlying data, including biotransformation rules and a curated database (MetXBioDB), are openly accessible through a web service, as a downloadable Java Library^91^ and on the NORMAN-SLE.^74^ A major update, BioTransformer 4.0, is expected soon but is not officially released at the time of writing. It introduces over 130 new reaction rules, a validation module that filters unrealistic metabolites based on similarity to known human metabolites, and a new abiotic metabolism module covering photolysis, chlorination, and ozonation reactions, partly derived from the CTS database. In the environmental metabolism module, the update improves SMIRKS string handling and fixes incorrect transformation rules that previously produced invalid metabolites.

A second option offering a variety of transformation algorithms is the Reaction Pathway Simulator module in the Chemical Transformation Simulator (CTS) by the U.S. EPA.^92^ It integrates various tools, such as EPISuite, the Toxicity Estimation Software Tool (T.E.S.T.), ChemAxon and OPEn structure–activity/property Relationship App (OPERA). CTS offers flexible input options (Name, SMILES, CAS, sketcher input). CTS employs defined reaction libraries that include generalized reaction schemes, specifying how a molecular fragment is modified by a particular transformation process. When a molecule is submitted, CTS compares its structure to the reactant side of these schemes in the libraries. If a match is found, the tool modifies the matched fragment while leaving the rest of the molecule unchanged. This mechanism is not unique to CTS, but rather the general principle of rule-based approaches. CTS prioritizes predicted TPs by ranking them based on transformation rates reported in scientific literature. Currently, CTS provides reaction libraries for abiotic hydrolysis, abiotic reduction, direct photolysis, spontaneous reactions (e.g., dehydration of geminal diols), human phase I metabolism, and both environmental and metabolic reactions of per- and polyfluoroalkyl substances (PFAS). Each reaction library includes schematic reactions and references to the scientific rules underlying the predictions. Additionally, CTS offers integration with other tools such as BioTransformer and enviPath Pathway Predictions, accessible through their respective APIs. While CTS has a GitHub repository (https://github.com/quanted/cts_app), much of its code relies on licensed software, limiting the creation of a fully independent clone. However, users can incorporate CTS into individual workflows via its REST API (https://qed.epa.gov/cts/rest/).

Another option to present here for TP prediction is the EAWAG-Biocatalysis/Biodegradation Database (BBD) Pathway Prediction System (PPS), which is also a rule-based, substructure searching, and atom-to-atom mapping prediction algorithm based on the biodegradation/biocatalysis database of the University of Minnesota.^93,94^ The 249 biotransformation rules are publicly accessible (http://eawag-bbd.ethz.ch/servlets/pageservlet?ptype=allrules) and typically include a scientific reference for each reaction. Reaction rules are also prioritized based on likelihood assigned by an expert panel to each reaction. This ranges from very likely and likely (e.g., spontaneous hydrolysis in water), possible for reactions that are common but not certain to occur in every system (e.g., transformation of a secondary alcohol to a ketone), to unlikely and very unlikely for reactions only very rarely catalyzed in bacteria or fungi (e.g., reductive dehalogenation). The BBD-PPS terminate its prediction once certain small compounds are reached (http://eawag-bbd.ethz.ch/servlets/pageservlet?ptype=termcompsview). These terminal compounds include two categories: (1) small, readily degraded molecules that do not undergo further transformation, and (2) dead-end compounds, often larger or halogenated, that are known to persist in the environment due to their resistance to microbial degradation. If a compound in category (1) is encountered, its biodegradation is not predicted further, but instead a link to a relevant Kyoto Encyclopedia of Genes and Genomes (KEGG)^95^ metabolic pathway is given. For compounds in category (2), no further transformation or KEGG pathway is offered. enviPath (envipath.org) expands the capabilities of the BBD-PPS with updated and more comprehensive reaction rules, an enhanced user interface, and integrated links to additional biochemical pathway databases, offering a more robust and user-friendly experience for exploring biotransformation pathways.^63^ While BBD-PPS advised caution with molecules over 1000 Da and excluded PFAS and highly fluorinated chemicals due to limited rule coverage, enviPath addresses these limitations. A recent addition is a dedicated PFAS (per- and polyfluoroalkyl substances) package,^96^ which includes curated microbial transformation pathways and trained reaction rules for selected fluorinated precursors. This targeted effort extends enviPath’s predictive reach toward highly persistent and environmentally relevant contaminants. Furthermore, enviPath’s open access database supports user contributions, enabling the continuous evolution of its predictive capabilities and the inclusion of diverse environmental conditions. This approach broadens the scope of chemicals that can be analyzed and improves the selectivity and reliability of the predictions.

Recently, the open-source platform patRoon,^21,84,85^ integrated several of these predictive techniques into a pipeline connecting *in silico* predictions with HRMS data. Alongside the tools already discussed, patRoon includes the PubChem/NORMAN-SLE transformation datasets as well,^65^ allowing users to systematically screen and annotate known and predicted TPs in their experimental data. This modular and extensible workflow enables researchers to efficiently prioritize and confirm TPs. Functionality for photolysis-related TP prediction and screening was added in 2025, further expanding patRoon’s ability to capture both biotic and abiotic transformation pathways.^21^ Through this integration, patRoon enhances the efficiency, reproducibility, and transparency of nontarget and suspect screening workflows.

As described above, enviPath is a highly curated predictive system specifically for environmental use cases, whereas CTS and BioTransformer offer environmental and additional metabolism functions. CTS also integrates abiotic reactions covering advanced treatment processes (functionality that is currently being developed in BioTransformer). Both CTS and BioTransformer integrate enviPath, while patRoon (a HRMS processing software) integrates all approaches and more. Thus, each approach offers significant overlap and the choice of which is the best in various scenarios may come down to user preferences.

## 5 Toxicological Assessment Tools

Unless otherwise specified, all tools discussed in this section (Table 1) are designed for organic compounds with well-defined molecular structures and do not support mixtures, substances of unknown or variable composition, nanomaterials, or polymers. These are general limitations of current QSAR and ML models due to the lack of consistent structural representation and training data for such complex substances.

A widely recognized predictive toxicity tool is the Estimation Program Interface, or EPISuite.^97^ EPISuite integrates various models to estimate physicochemical properties and the Ecological Structure Activity Relationships (ECOSAR) predictive models, which are also available separately. ECOSAR models estimate aquatic ecotoxicity based on equations derived from experimental data, allowing for the evaluation of several endpoints across multiple organisms within the aquatic food chain. These include green algae (72 or 96 h tests), *Daphnia* (48 h tests), and fish (96 h tests) for both acute lethality and chronic values. The user interface supports batch mode processing. While EPISuite results are validated internally, limited availability of the training and validation datasets hamper independent assessment of the applicability domains (Table 1). Recent studies highlighted limitations for phytotoxins^98^ and those with atypical functional groups, particularly for fluorinated and phosphorus-containing compounds.^99^

**Table 1:** Overview of the *In Silico* Tools Described in This Article, Their Included Models/Endpoints, Data Accessibility and Applicability Domain Estimation (Further Details Are Given in the Main Text)

Tool	Main focus	Included models	Training dataset accessible	Applicability domain provided
EPISuite^97^	physicochemical properties, ecotoxicology	multiple QSARs and ECOSAR	limited	not for all models
ToxTree^100^	toxicological hazard screening	cramer rules, verhaar scheme, Benigni/Bossa rules	yes	rule-based
T.E.S.T.^101^	ecotoxicology, human toxicity	QSARs	yes (ECOTOX database)	yes
OPERA^102^	physicochemical properties, human endocrine activity	CERAPP, CoMPARA, CATMoS	yes	yes
VEGA-QSAR^103^	physicochemical properties, ecotoxicology, toxicology, environmental fate	>100 models from CAESAR, OPERA, ECOSAR, etc.	yes	yes
TRIDENT^104^	ecotoxicology	deep learning transformer model	yes (Github)	yes
NR-ToxPred^105^	human endocrine activity	9 receptor models	yes	yes

A free open-source rule-based tool to predict the toxicological hazard of chemicals is ToxTree.^100^ It applies various decision tree models incorporated into the concept of threshold of toxicological concern to assess the so-called Cramer class of a chemical substance to estimate its relative toxic hazard. ToxTree evaluates chemical structures against a set of predefined rules or structural alerts to determine potential hazards, which is useful for initial hazard assessment in chemical safety evaluation. ToxTree offers multiple classification schemes, including Cramer decision tree for oral toxicity classification, Verhaar scheme for mode of toxic action of organic chemicals, Benigni/Bossa rule-based mutagenicity and carcinogenicity alerts. The tool provides transparent and interpretable results, as each classification follows explicit mechanistically relevant rules. ToxTree supports batch processing and accepts SMILES, MOL, and SDF files as input formats.

The Toxicity Estimation Software Tool (T.E.S.T.) incorporates the Computer Assisted Evaluation of industrial chemical Substances According to Regulations (CAESAR) model for developmental toxicity as well as carcinogenicity and mutagenicity models, also implemented in VEGA-QSAR. The open-access tool also incorporates models for the prediction of endpoints for fathead minnow LC_50_ (96 h), *Daphnia magna* LC_50_ (48 h), *tetrahymena pyroformis* IGC_50_, oral rat toxicity (LD_50_), and bioaccumulation factor for fish.^106–110^ T.E.S.T. uses several ML models along with conventional QSAR methods and accepts CAS, SMILES, name, InChI, InChIKey, DTXSID, or sketcher input. Batch mode processing is supported (txt, SMILES, SDF). Compounds must have defined structures and fall within the model’s molecular weight range (≤2000 Da). The outputs are offered in different formats (csv, excel or html). The batch mode processes multiple chemicals for only a single end point at one time. Model specific validation results for T.E.S.T. are documented in the User’s Guide, while all experimental toxicity data used for model development originates from the publicly available ECOTOX database, allowing for independent evaluation and further analysis.

The OPEn qsaR App (OPERA) includes predictions for estrogenic activity from the Collaborative Estrogen Receptor Activity Prediction Project (CERAPP),^111^ Androgenic activity from the Collaborative Modeling project for Androgen Receptor Activity (CoMPARA),^112^ as well as the acute oral systematic toxicity from the Collaborative Acute Toxicity Modeling Suite (CATMoS),^113^ and predictions of physicochemical properties such as acid dissociation constant, octanol–water partitioning coefficient and distribution constant for nonionizable compounds.^114–116^ OPERA is open source (https://github.com/kmansouri/OPERA) and can be used locally with or without graphical user interface. It is included in several open resources, including the U.S. EPA CompTox Chemicals Dashboard^117^ and as extension in the QSAR Toolbox.^118,119^ OPERA allows batch mode processing with various input formats (SMILES, SDF, MOL, CASRN, DTXSID, DTXCID, InChIKey) and returns a list of molecule IDs, predictions, the applicability domain and an accuracy assessment.^102,120^ One of OPERA’s key strengths is its applicability domain assessment, based on structural similarity measures, leverage-based methods, and distance-to-model calculations, to assess how closely a given compound aligns with its training data set.

VEGA-QSAR is an open-access tool integrating over 100 predictive models, combining various QSAR-based toxicological, environmental, and physicochemical assessments. It incorporates models from CAESAR,^121,122^ OPERA, EPI Suite,^102,123,124^ and others,^100,125^ supporting regulatory and environmental applications. VEGA has put emphasis on ensuring that the models generate transparent and reproducible results, providing model guides, test and training datasets accessible in the standalone application (Figure 1), facilitating screening of these datasets and checking the applicability of the respective model. It supports different standard formats used in the chemical domain, including SMILES and SDF. Batch mode is available, including multiple model selection. VEGA can also be used for read-across approaches without involving QSAR models.^126^

**Figure 1:**
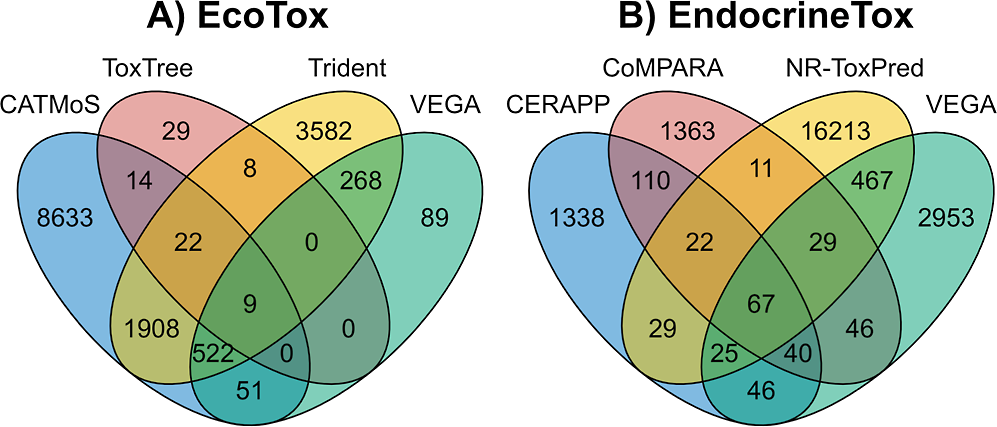
Number of compounds included in the training and test datasets for (A) ecotoxicological endpoints (EcoTox) and (B) endocrine endpoints (EndocrineTox). For VEGA in A), the datasets used were fish acute LC_50_ SarPy/IRFMN, *Daphnia magna* LC_50_ IRFMN, and algae acute EC_50_ IRFMN. For VEGA in B), the datasets used were androgen receptor-mediated effect (IRFMN/CoMPARA), estrogen receptor-mediated effect (IRFMN/CERAPP), and estrogen receptor relative binding affinity (IRFMN). Datasets were merged using SMILES and CAS numbers when available.

A recent model for ecotoxicological end point prediction is the deep learning model TRIDENT,^104^ which is based on the transformer architecture. TRIDENT predicts two toxicity endpoints, EC_50_ and EC_10_, for three species groups (algae, aquatic invertebrates and fish) and a variety of effects. The web-service version uses SMILES (https://trident.serve.scilifelab.se/) and allows, depending on the combination of end point and species group, predictions for mortality, intoxication, population, reproduction, and growth. The code, full model and data set used to develop the model, consisting of almost 150 000 experimental data for 6657 unique chemicals (Figure 1), are available online (https://github.com/StyrbjornKall/TRIDENT). The training data set includes a large fraction of charged chemicals (∼25%), including inorganic compounds such as NiF_2_, FeCl_3_, Fe_2_O_3_, PbSO_4_ and PdO. While most tools exclude such compounds, TRIDENT’s training data include a number of organometallics like hydroxy-methylmercury, expanding its coverage slightly beyond typical mode. TRIDENT outperformed three existing models (ECOSAR, VEGA, and T.E.S.T.) for most endpoints, except algae EC_50_.^104^

In addition to OPERA, the ML model NR-ToxPred offers *in silico* predictions of endocrine activity by assessing ligand binding to nine human nuclear receptors (e.g., androgen, estrogen α/β, progesterone). Based on a public data set of ∼15,000 entries (Figure 1), the model provides binary predictions (active/inactive, binding/nonbinding) along with sensitivity, specificity, and applicability domain estimates using the Tanimoto similarity measure.^127^ Unlike OPERA, NR-ToxPred does not distinguish between agonists and antagonists, lacks uncertainty quantification, and is limited to organic compounds. Although the model code is not public, the tool is accessible via a user-friendly web interface (http://nr-toxpred.cchem.berkeley.edu/) and supports batch prediction with CSV input and receptor binding site visualization.

There are numerous other toxicity prediction models available, targeting specific organisms, endpoints, or effects, as detailed elsewhere.^119,128–135^ The online chemical modeling environment (OCHEM) can be used to run available models to screen compounds for structural alerts for (eco)­toxicological endpoints, and also provides the opportunity to create new QSAR models based on the experimental data in the database.^136–139^ Two research groups have recently developed algorithms to estimate ecotoxicity endpoints from HRMS fragment data.^140,141^ Such approaches could facilitate chemical risk assessment from chemical screening data and provide further insights into mixture toxicity assessment. Additionally, conventional dose–response models may fall short in accounting for continuous low-level exposure or the specific toxicokinetic behavior of highly persistent or bioaccumulative substances.^142^ For example, differences in compound distribution, such as accumulation in fatty tissues versus protein binding, can significantly affect internal exposure and toxicodynamics. The integration of pharmacokinetic-pharmacodynamic modeling, which assesses the relationship between chemical exposure and biological response over time, could enhance prediction accuracy by incorporating absorption, distribution, metabolism, and excretion dynamics. These models are particularly relevant for widespread contaminants and extremely persistent chemicals, where chronic exposure scenarios may be more representative of real-world environmental conditions. In cases where a hypothesis of the specific mode of toxic actions exists, this can be confirmed and its understanding deepened via *in silico* tools, such as molecular docking or molecular dynamic simulations with free energy perturbations, as discussed recently.^46^ These techniques require more bioinformatics and command line skills than the previously described approaches, but could initiate the development of adverse outcome pathways and by that contribute for example to a computational ecotoxicity assay.^45^

## 6 Remarks for Future

*In silico* approaches for TP and toxicity predictions are beneficial to researchers and legislators in providing additional acquisition of toxicity-related information on TPs. Advances in ML and computational power have made it easier to develop predictive models; however, meaningful improvements in prediction accuracy depend on robust validation methods and well-defined criteria. While models are becoming more sophisticated, many suffer from overfitting, heavy bias, or poor generalizability due to for example limited and biased training datasets. A clear understanding of estimation methods and their appropriate application is therefore critical. Beyond ensuring alignment with best-practice guidelines,^143–146^ we propose four distinct levels of confidence (Figure 2) to be reported for enhancing both interpretability and reliability of TP predictions.

1. High confidence (validated and reliable)

**Figure 2:**
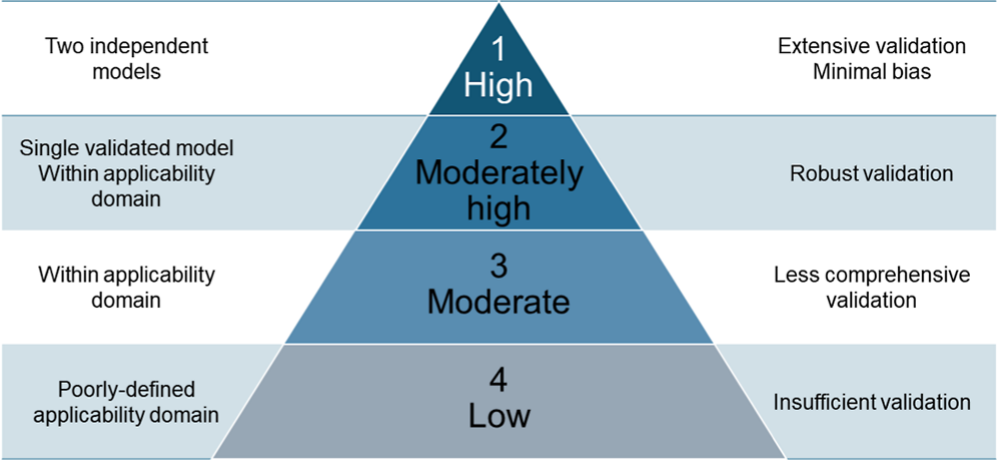
Schematic visualization of the confidence levels including defining criteria.

Two or more independent models with well-defined applicability domains and extensive validation across diverse datasets. Minimal bias, strong generalization across chemical classes, and mechanistic support from rule-based models with literature backing up.

*Example: Prediction of acute fish toxicity for 4-nitrophenol using VEGA-QSAR and TRIDENT. The compound falls within the applicability domain of both models and is included in their training datasets. This direct inclusion greatly enhances the reliability and confidence in the predicted toxicity values.*

2. Moderately high confidence (reliable but less broadly validated)

Single validated model with a well-defined applicability domain, robust validation, and transparent methodology (e.g., public datasets). Rule-based models supported by mechanistic plausibility but lacking experimental confirmation for similar chemical compounds.

*Example: Prediction of estrogen binding potential of bisphenol S using the OPERA platform (CEARPP model for estrogenicity). The prediction is within the model’s applicability domain and supported by robust validation and clear mechanistic relevance. Although no experimental data for bisphenol S are present in the model’s training data set, its close analogue bisphenol A is well represented, providing additional support and resulting in moderately high confidence in the prediction.*

3. Moderate confidence (limited generalization)

Predictions within the applicability domain but with less comprehensive validation or uncertain generalization beyond specific datasets. Rule-based models relying on mechanistic assumptions but lacking empirical validation for the relevant chemical class.

*Example: Prediction of acute Daphnia toxicity for ciprofloxacin using the VEGA-QSAR model is of moderate confidence. While the compound’s broad structure may be technically within the model’s applicability domain, ciprofloxacin and related fluoroquinolone antibiotics are not represented in the VEGA training set, and the model has not been comprehensively validated for this chemical class. Therefore, there is uncertainty in the prediction’s reliability for antibiotics with ionizable and zwitterionic properties.*

4. Low confidence (uncertain or limited reliability)

Predictions from models with poorly defined applicability domains, insufficient validation, or high uncertainty in extrapolation.

*Example: Prediction of acute algal toxicity for novel silicon-containing compound using T.E.S.T model. However, because organosilicons are not represented in the training data and the applicability domain for this class is poorly defined, the reliability of the prediction is considered low confidence.*

Following the European Food Safety Authority (EFSA) guidelines, the use of two independent QSAR models confirming predictions is recommended,^147,148^ where independence refers to differing training datasets or algorithms (rule-based *vs* statistical). Both models should be of high to moderate-high confidence. Most models do not account for mixture toxicity effects (e.g., additive or synergistic effects of chemicals).^149^ Furthermore, environmental conditions can vary and should be considered for ionic and ionizable chemicals, as these factors can govern e.g., the partitioning in environmental systems.^150,151^ The validation of most predictive toxicology models using novel compounds (not included in any test or training data set) with different modes of action is of high interest to experimentally validate accuracy and precision of the models.

While this article highlights the potential for computational TP and toxicity prediction methodologies to support research and enhance risk assessments of TPs, predictive reliability remains variable across different chemical classes due to uneven data coverage. A concerted community effort on generating and sharing relevant data for greater portions of the “chemical space”, rather than generating yet more data for compounds very similar to existing data, would help expand the applicability domains—and thus increasing the usefulness of these computational approaches immensely. Additionally, TPs formed during water treatment processes (e.g., advanced oxidation processes like ozonation) are gaining attention, especially in light of the recast EU wastewater treatment directive (EU 2024/3019).^152^ Despite their growing environmental relevance, these treatment-derived TPs are often underrepresented or unsupported in current *in silico* tools, although recent developments are striving to cover this gap. Expanding the underlying experimental data collections as well as model rules/coverage to include these TPs would help align computational assessments more closely with real-world transformation pathways and support regulatory needs.
